# ENHANCE: a comparative prospective longitudinal study of cognitive outcomes after 3 years of hearing aid use in older adults

**DOI:** 10.3389/fnagi.2023.1302185

**Published:** 2024-01-31

**Authors:** Julia Z. Sarant, Peter A. Busby, Adrian J. Schembri, Christopher Fowler, David C. Harris

**Affiliations:** ^1^Department of Audiology and Speech Pathology, The University of Melbourne, Melbourne, VIC, Australia; ^2^Cogstate, Melbourne, VIC, Australia; ^3^The Florey Institute of Neuroscience and Mental Health, Melbourne, VIC, Australia; ^4^Department of Economics, The University of Melbourne, Melbourne, VIC, Australia

**Keywords:** hearing loss, dementia, hearing aids, cognitive performance, risk factor, delay, intervention

## Abstract

**Background:**

With an aging population, the prevalence of hearing loss and dementia are increasing rapidly. Hearing loss is currently considered the largest potentially modifiable risk factor for dementia. The effect of hearing interventions on cognitive function should therefore be investigated, as if effective, these may be successfully implemented to modify cognitive outcomes for older adults with hearing loss.

**Methods:**

This prospective longitudinal observational cohort study compared outcomes of a convenience sample of prospectively recruited first-time hearing aid users without dementia from an audiology center with those of community-living older adults participating in a large prospective longitudinal cohort study with/without hearing loss and/or hearing aids. All participants were assessed at baseline, 18 months, and 36 months using the same measures.

**Results:**

Participants were 160 audiology clinic patients (48.8% female patient; mean age 73.5 years) with mild–severe hearing loss, fitted with hearing aids at baseline, and 102 participants of the Australian Imaging, Biomarkers and Lifestyle Flagship Study of Aging (AIBL) (55.9% female patient; mean age 74.5 years). 18- and 36-month outcomes of subsets of the first participants to reach these points and complete the cognition assessment to date are compared. Primary comparative analysis showed cognitive stability for the hearing aid group while the AIBL group declined on working memory, visual attention, and psychomotor function. There was a non-significant trend for decline in visual learning for the AIBL group versus no decline for the hearing aid group. The hearing aid group showed significant decline on only 1 subtest and at a significantly slower rate than for the AIBL participants (*p* < 0.05). When education effects on cognitive trajectory were controlled, the HA group still performed significantly better on visual attention and psychomotor function (lower educated participants only) compared to the AIBL group but not on working memory or visual learning. Physical activity had no effect on cognitive performance trajectory.

**Conclusion:**

Hearing aid users demonstrated significantly better cognitive performance to 3 years post-fitting, suggesting that hearing intervention may delay cognitive decline/dementia onset in older adults. Further studies using appropriate measures of cognition, hearing, and device use, with longer follow-up, are required.

## Introduction

With an aging population, the global burden of dementia is expected to increase significantly over the next few decades. Given limited pharmaceutical treatments, an increased effort has more recently been made to identify potentially modifiable risk factors for this disease. There is now substantial evidence that hearing loss (HL) is associated with accelerated cognitive decline (Albers et al., 2015; Loughrey et al., 2018; Ray et al., 2018; Huang et al., 2023), with the Lancet Commission on dementia identifying it as the largest known potentially modifiable risk factor, accounting for 8–9% of dementia risk (Livingston et al., 2020). The estimated risk of incident dementia for people with HL compared to that of those with normal hearing is estimated to be doubled for those with mild loss, almost tripled for those with moderate loss, and almost five times greater for those with severe loss (Lin et al., 2011). Despite being highly prevalent in older adults, with over 58% of disabling HL experienced by adults aged 60 years and older (WHO, 2021b), there is a lack of awareness of this risk and of the other comorbidities associated with HL (e.g., falls, depression, and earlier mortality; Australian Hearing, 2017), with less than 11% of adults with disabling HL worldwide estimated to be hearing aid (HA) users (Bisgaard et al., 2021). Other physical and psychiatric health conditions that are also risk factors for cognitive decline and/or are associated with HL include cardiovascular disease, stroke, cancer, diabetes, depression, and anxiety (see Livingston et al., 2020 for a review).

Several causal mechanisms for the association between HL and dementia have been proposed (for reviews of these, see Uchida et al., 2014; Fulton et al., 2015; Griffiths et al., 2020). Three hypotheses suggest mechanisms which could potentially be modified through hearing intervention. These mechanisms include HL causing decreased auditory stimulation of cognitive processing and subsequent changes in brain structure and function; cognitive resource re-allocation and depletion due to the need for greater resources for listening to and processing reduced auditory stimuli; and reduced environmental stimulation and social participation due to HL, with subsequent psychological sequelae such as loneliness and depression, causing changes in brain structure and function. If any of these hypothesized mechanisms are causal, it is likely that they are not mutually exclusive. Furthermore, the relationship between hearing and cognition may be bidirectional.

Given HL often precedes the onset of dementia by at least 5–10 years, if hearing intervention is effective in addressing one or more of the hypothesized mechanisms, there may be a potential window of opportunity for delaying dementia onset. It is estimated that if this is the case, delaying dementia onset for even 1 year could decrease its global prevalence by 10% (Peracino, 2015), a significant outcome. Although some observational studies have reported positive effects of HA use on cognition in older adults (Mahmoudi et al., 2019), and there is evidence of neural plasticity after HA use (Giroud et al., 2017; Glick and Sharma, 2020), there have been mixed outcomes. Recent systematic reviews have also come to different conclusions regarding the effect of HA use on cognitive outcomes and the quality of the evidence. Sanders et al. (2021) examined cognitive outcomes by cognitive domain and global function, concluding the effects of HA use on cognition are unclear, with more null than positive findings, and overall poor quality of studies. Yang et al. (2022) reported significant improvements in observational studies on some tests of global function, working memory, and executive function but that more studies showed no significant effects overall, while meta-analysis of randomized control trial (RCT) results showed no effect of HAs on cognitive function. High risk of bias and many methodological limitations were noted. Yeo et al. (2022) concluded that HA use was associated with a decreased risk of cognitive decline but included only studies measuring general cognitive function. Although the focus in the reviews was on improved cognitive function with HA use, it should also be considered that stability or lack of decline is an important positive outcome that would contribute toward delay of dementia onset.

As noted in the systematic reviews, a significant methodological limitation of many studies is the use of inappropriate cognitive performance assessment methods, including the use of insensitive cognitive screening tests, test administration to people with HL using auditory instructions, and making either unspecified or non-standardized changes to tests. Even the use of written instructions and best practice in test administration is associated with worse cognitive outcomes for people with HL on both auditory-only and non-auditory only tests, who score more poorly and are much less likely to complete cognitive testing, either due to misheard instructions or increased cognitive load/fatigue. Missing data in these cases is reported to underestimate the HL–cognition relationship by 30% (Deal et al., 2021). Other significant methodological issues include small sample size, self-report or no measurement of HL and/or device use, lack of measurement of intervention benefits, and lack of a control/comparative sample (Amieva et al., 2015; Deal et al., 2015; Mahmoudi et al., 2019; Bucholc et al., 2021). Most studies had short follow-up periods of only 6–12 months, so the long-term effects of HA use on cognition are unknown. Change in hearing over time was not usually measured, precluding the longitudinal examination of any relationship between changes in hearing and cognition.

The first large-scale RCT on the effect of HA use on cognitive performance over 3 years has recently been published (ACHIEVE; Lin et al., 2023). The ACHIEVE study compared hearing intervention with HAs with a healthy aging education program in healthy community volunteers and participants of a 3-year longitudinal study of cardiovascular health (ARIC) who were at higher risk for dementia. This study provides an important contribution to the evidence. However, it cannot answer the question of causality in HL and dementia due to methodological limitations. The trial’s primary outcome was no significant difference in global cognition outcomes between aided and unaided groups. However, a secondary analysis showed HA use significantly reduced the 3-year global rate of cognitive decline in the ARIC group by 48% compared to the ARIC control group. It was posited that the null primary outcome could have been due to inadequate length of follow-up. A significant methodological issue in this trial, as for many, is potential confounding of the cognitive data through the use of auditorily administered cognitive assessments to participants with HL, as those with worse hearing/no HAs would be less able to hear instructions correctly and may experience greater cognitive load in undertaking the cognitive tasks, even with written instructions for some assessments and best practice (Saunders et al., 2018; Kim et al., 2023). Given this, it is unlikely that data collected over the telephone during COVID using a reduced cognitive battery that were included in the secondary analysis were comparable to the data used for the primary analysis. Furthermore, there was no objective measurement of either intervention exposure [HA use is widely reported to be inflated for self-report, (Laplante-Lévesque et al., 2014; Solheim and Hickson, 2017)] or intervention efficacy (significantly improved hearing/speech perception with HAs).

Given the above, further randomized control trials and prospective observational longitudinal studies of samples at risk for dementia that address the current methodological limitations in the evidence are still needed to better understand the association between HL and dementia. These would ideally assess not only cognitive function as the primary outcome but also cognitive impairment/dementia outcomes both during the study and at drop-out, in order that dementia conversion rates that may be attributable to HL are measured.

The current Evaluation of Hearing Aids and Cognitive Effects (ENHANCE) study investigated the effect of HA use on cognition and other outcomes over 3 years in older adults with age-related HL, controlling for baseline key dementia risk factors and comparing outcomes with those of a representative group of community-living older adults with either normal hearing or untreated HL as would be expected in the general community. HL, HA benefits (intervention effectiveness), and device use (compliance) were objectively longitudinally assessed, and cognition was assessed using a non-auditorily presented tool to avoid confounding due to HL on understanding of test instructions. This study design overcomes many of the limitations of previous studies, the most important of which are the visual presentation of a highly sensitive non-screening cognitive performance battery to avoid confounding of cognitive data by auditory administration of instructions to people with hearing loss, objective assessment of hearing loss, device use, and device benefits, and the inclusion of an untreated comparative group representative of the community of older adults and who were assessed using the same protocols. Additionally, the statistical analyses included controls (fixed effects) not only for all differences between the two groups at baseline but also for known differences across the cognitive trajectory, unusual for most observational studies. Initial 18-month follow-up data and methodology in a smaller HA user sample showed either significant improvement or stability in cognitive function across different subtests for older adult HA users (Sarant et al., 2020).

## Materials and methods

### Study design and participants

Adults aged ≥60 years who attended a metropolitan audiology clinic, with diagnosed HL [mean better ear Pure Tone Average (PTA4) of 20dBHL or greater; (WHO, 2021a)], no previously diagnosed or suspected cognitive impairment, who passed the cognitive screening assessment (see below), and had no language difficulties that prevented them from completing the assessment protocol were eligible to participate in the study in the intervention group. A total of 160 naive HA users were cognitively assessed at baseline before HA fitting and subsets of this group were assessed at 18 and 36 months post-HA fitting. After HL diagnosis in the initial appointment and a decision to trial HAs, standard HA fitting clinical procedure was followed. This involved prescription of HAs of different brands by audiologists dependent on type and degree of HL and participant preferences using the NAL-NL2 prescription (Keidser et al., 2011) unless clients preferred otherwise. The suitability of participant HA fittings was reviewed, in line with standard clinical practice, 2–4 weeks after fitting to determine the appropriateness of the fitting and to make any required adjustments to HA settings, with further reviews as necessary based on participant preferences and standard minimum yearly follow-up, including repeated HL assessment.

Outcomes for the intervention (HA) group were compared with those of a sample of participants with untreated HL and normal hearing recruited from a large longitudinal cohort study of community-living older adults (Australian Imaging, Biomarker and Lifestyle Flagship Study of Ageing; AIBL; Fowler et al., 2021) at the same time intervals and using the same assessment battery (except for device use, as AIBL participants did not use a device). The AIBL study, launched in 2006, is a large Australian prospective cohort study aiming to investigate the natural history of Alzheimer’s disease from preclinical onset to the development of dementia. The study databank includes biospecimens, brain imaging, and clinical and cognitive performance data, which will be used to determine which biomarkers, cognitive characteristics, and health and lifestyle factors are predictive of the development and progression of Alzheimer’s disease. This sample included older adults both without HL and with HL who did not use HAs, as would be expected in a representative sample of the general population. Demographic and audiological data for all participants are summarized in Table 1.

**Table 1:** Demographic and audiometric characteristics of participants at baseline, 18-, and 36-month follow-up.

	HA participants			AIBL participants			HA versus AIBL (value of *p*)		
	Baseline	18 months	36 months	Baseline	18 months	36 months	Baseline	18 months	36 months
*Age (years)*									
*n*	160	61	54	102	49	18			
Mean	73.5	75.09	75.71	74.44	75.35	77.24	0.074	0.735	0.13
Median	73.5	74.1	75.6	74.5	74.9	77.1			
SD	4.3	4.2	3.9	4	3.9	3.5			
Min	67	68.9	70.2	67	68.4	71.4			
Max	86.9	88.3	85.2	84.8	83	84			
*Better Ear PTA4^a^*									
*n*	160	61	54	102	38	17			
Mean	31.41	33.71	36.57	21.27	22.3	23.53	**0.000**	**0.000**	**0.000**
Median	30	33.8	37.5	21.2	21.2	23.8			
SD	9	8.2	8.3	8.7	9.2	8.3			
Min	12.5	15	20	3.8	6.2	8.8			
Max	63.8	55	56.2	45	46.2	37.5			
*Normal Hearing^b^*									
*N*	160	61	54	102	38	17			
No. (%)	16 (10)	2 (3.3)	1 (1.9)	47 (46.1)	18 (47.4)	8 (47.1)	**0.000**	**0.000**	**0.002**
*Female participant*									
*n*	160	61	54	102	49	18			
No. (%)	78 (48.8)	26 (42.6)	25 (46.3)	56 (54.9)	28 (57.1)	10 (55.6)	0.333	0.133	0.509
*Education >12 years*									
*n*	149	61	54	102	49	18			
No. (%)	125 (83.9)	52 (85.2)	45 (83.3)	74 (72.5)	29 (59.2)	11 (61.1)	**0.036**	**0.003**	0.098
*Diabetes*									
*n*	149	60	52	98	33	12			
No. (%)	15 (10.1)	3 (5)	1 (1.9)	5 (5.1)	2 (6.1)	2 (16.7)	0.138	0.835	0.221
*Depression*									
*n*	149	60	52	98	33	12			
No. (%)	25 (16.8)	10 (16.7)	8 (15.4)	7 (7.1)	0 (0)	2 (16.7)	**0.018**	**0.001**	0.918
*Anxiety*									
*n*	149	60	52	97	34	12			
No. (%)	29 (19.5)	12 (20)	7 (13.5)	7 (7.2)	3 (8.8)	1 (8.3)	**0.004**	0.123	0.600
*Falls*									
*n*	149	60	52	98	34	12			
No. (%)	13 (8.7)	7 (11.7)	10 (19.2)	8 (8.2)	3 (8.8)	4 (33.3)	0.877	0.662	0.370
*Cardiovascular condition*									
*n*	149	60	52	97	34	13			
No. (%)	73 (49)	36 (60)	32 (61.5)	45 (46.4)	17 (50)	7 (53.8)	0.691	0.357	0.635
*Retired*									
*n*	149	60	53	98	34	13			
No. (%)	121 (81.2)	48 (80)	44 (83)	87 (88.8)	32 (94.1)	13 (100)	0.097	**0.036**	**0.002**
*Ever smoker*									
*n*	149	23	47	47	26	9			
No. (%)	65 (43.6)	11 (47.8)	20 (42.6)	17 (36.2)	10 (38.5)	1 (11.1)	0.365	0.519	**0.031**
*1 Apolipoprotein E (APOE) ε4 allele*									
*n*	82	57	53	101	48	18			
No. (%)	24 (29.3)	20 (35.1)	17 (32.1)	29 (28.7)	17 (35.4)	5 (27.8)	0.935	0.972	0.736
*2 Apolipoprotein E (APOE) ε4 alleles*									
*n*	82	57	53	101	48	18			
No. (%)	1 (1.2)	1 (1.8)	1 (1.9)	1 (1)	1 (2.1)	0 (0)	0.884	0.904	0.322
*Living alone*									
*n*	149	61	54	99	48	17			
No. (Pct)	37 (24.8)	13 (21.3)	9 (16.7)	24 (24.2)	11 (22.9)	4 (23.5)	0.916	0.843	0.565
*Activity*									
*n*	149	59	53	102	49	18			
Mean	4596.5	4846.72	5715.55	2896.94	2826.19	2689.17	**0.000**	**0.012**	**0.003**
Median	3,402	4,239	4,200	2030.2	1,262	1704			
S.D.	4478.7	3987.2	5084.8	2965.8	4180.9	2911.7			
Min	0	438	297	0	0	0			
Max	34,008	25,560	24,906	13,086	20,067	8,163			

Bold denotes significant differences (*p* < 0.05; significant at the 5% confidence level). SD, standard deviation. ^a^PTA4 ≤ than 20 dB hearing level. ^b^International Physical Activity Questionnaire (IPAQ); mean met minutes per week.

### Outcome variables

The primary outcome of this study was cognitive performance. Other outcomes included audiometrically measured hearing thresholds, speech perception benefits, and device use.

#### Cognitive assessment using standardized visually presented assessment battery

The Mini-Mental State Examination (MMSE; Folstein et al., 1975) was used to screen for dementia at baseline. Cognition was thereafter assessed using the Cogstate computerized Brief Battery (Falleti et al., 2006; Maruff et al., 2009, 2013), administered by audiologists trained and supervised by a neuropsychologist. The Cogstate Brief Battery was developed for repeated assessment of cognitive performance, is highly reliable (test–retest reliability for each subtest ranges between 0.84 and 0.94), and facilitates minimal practice effects (Collie et al., 2003; Falleti et al., 2006). It is visually presented and therefore highly suitable for use with people with HL. Assessments used include psychomotor function (Detection test), attention (Identification test), working memory (One Back test), and visual learning (One Card Learning test). Speed and accuracy of responses were transformed on a centralized platform to yield normalized data distributions (Falleti et al., 2006; Maruff et al., 2009). Cogstate measures of information processing speed, attention, and memory have been shown to be highly sensitive to cognitive dysfunction and longitudinal cognitive decline in older adults (Lim et al., 2012; Maruff et al., 2013). Risk of bias regarding results was minimal, as after training, participants completed the assessment in a quiet room alone, and their de-identified results were automatically uploaded to the centralized Cogstate platform for automated scoring.

For each task, the speed and accuracy of each response are recorded. Also for each task, a single performance measure is selected on the basis it is derived from a normal data distribution, has an unrestricted range, no floor or ceiling effects, and has good reliability, stability, and sensitivity to change (Frederickson et al., 2010; Hammers et al., 2011). In a sample that is not entirely cognitively impaired, according to Cogstate protocol, psychomotor function (detection), visual attention (Identification), and working memory (One Back) are scored based on reaction time in milliseconds (speed); therefore, lower scores indicate better cognitive performance on these tasks. Visual learning (One Card Learning) is scored based on the proportion of correct answers (accuracy) and is reverse-scored, with higher scores indicating better cognitive performance. Primary outcome scores (raw scores) and not z-scores were used in this study to examine the relationship between age and cognitive performance, as this is not possible once raw data have been converted into a z-score, as these are standardized for age.

During the COVID pandemic when in-person visits were not allowed, sanitized laptops were delivered by clinicians to participant homes. Clinicians remained outside the homes in their cars to provide technical or instructional assistance if needed and to collect the laptops when the assessments were completed.

The Cogstate Brief Battery is not a diagnostic measure but is designed for profiling cognitive performance and change over time in people with and without dementia and was used for this purpose in the current study. Dementia outcomes were determined via a yearly medical history, in which participants and their significant others were asked whether they had received a medical diagnosis of cognitive impairment (i.e., mild cognitive impairment or dementia).

### Hearing loss and objective speech perception

HL was objectively assessed by an audiologist in a sound-proof booth or quiet room using gold-standard audiometric practice—pure tone audiometry (Kiely et al., 2012). Audiometric assessment included air and bone conduction thresholds, speech discrimination assessment, and tympanometry. Four-frequency pure tone averages (PTA4s; average of hearing thresholds at 500, 1,000, 2,000, and 4,000 kHz) were calculated, with a PTA4 of greater than 20 dB hearing level (HL) defined as HL, in accordance with World Health Organization criteria (WHO, 2021a). For descriptive (but not statistical) purposes, degree of HL was categorized using average PTA4s as normal (−10–20 dBHL), mild (21–40 dBHL), moderate (41–70 dBHL), and severe (71–90 dBHL).

Speech perception was assessed using recorded consonant–vowel–consonant (CVC) monosyllabic words (50-word lists; scored for words and phonemes correct) presented at 65dBSPL in quiet in the left ear, right ear, and binaurally unaided at baseline and in the best aided condition for participants post-device fitting. Speech reception threshold (SRT) testing was conducted using 20 Bamford-Kowal-Bench-like sentence lists presented at 65 dBSPL in 4-talker babble background noise, with variable noise level dependent on sentence scores. Speech and background noise were presented 1 meter in front of participants via a single speaker in free field. The non-test ear was masked in the unilateral listening conditions using white noise (30 dB above the average of the 1 and 2 kHz thresholds). The mean word score in signal-to-noise ratio was used to calculate speech in noise perception for the right ear, left ear, and binaurally. The final score indicated the signal-to-noise ratio at which 50% of the key words were correct.

### Medical health history

A detailed health and medical history was taken at baseline and updated at each follow-up point. This included family history of neurological illness and mental health problems, a personal health history including falls, cardiovascular health, diabetes, smoking, illicit drug, and medication use. Participants were classified as having a cardiovascular condition if they reported diagnosis of one or more of hypertension, angina, myocardial infarction, or stroke.

### Genetic screening

DNA genotyping using saliva samples taken at baseline was used to identify carriers of the apolipoprotein (APOE) ε4 allele, the strongest known genetic risk factor for late-onset Alzheimer’s dementia (Hunsberger et al., 2019; Emrani et al., 2020).

### Anxiety and depression

The Hospital Anxiety and Depression Scale (HADS; Zigmond and Snaith, 1983), designed for use with people who have physical health problems, was used to measure self-reported levels of anxiety and depression. Reported specificity and sensitivity for anxiety and depression are 0.78/0.9 and 0.79/0.83, respectively.

### Health and lifestyle

The International Physical Activity Questionnaire long form (Craig et al., 2003) was used to estimate levels of physical activity. The IPAQ includes four domains: during transportation, at work, during household and gardening tasks, and during leisure time, including exercise and participation in sport. Retirement status was also recorded at each assessment point.

Living arrangements as reported by participants were recorded at baseline and at each follow-up assessment.

### Statistical analysis

The full HA sample (*n* = 218) had a minimum age of 60.1 years, while the AIBL sample (*n* = 102) had a minimum age of 67.0 years. For comparative analysis with the AIBL group, the HA sample was reduced to include only participants with age at least 67 years (the age of the youngest AIBL participant), giving a HA sample size of 160 which was comparable to the AIBL sample with respect to age distribution (*p* = 0.074). This ensured that the two samples had “common support” with respect to age, that is, participants were present in each of the two samples throughout the restricted age distribution. Data for two AIBL participants were excluded from the analysis due to not all cognitive subtests being attempted. There were no missing data for HA participants.

Data were analyzed using R statistical software, version 4.2.2 (R Project for Statistical Computing; R Core Team, 2022).

Panel data multivariable regression modeling was used to quantify the average differences in outcomes between the HA and AIBL participant groups. The specification was as follows:


(1) $$Y_{i,t}=\alpha_{i}+\beta_{1}\;\left({\mathrm{AIBL}}_{i}\times {\mathrm{Years}}_{i,t}\right)+\beta_{2}\;\left({HA}_{i}\times {\mathrm{Years}}_{i,t}\right)+U_{i,t}\text{.}$$

where Y*_i,t_* is the outcome value for participant *i* at time *t* = 0, 1, and 2 (baseline, 18 months, and 36 months, respectively), α*_i_* is participant-specific time-invariant fixed effects, Years*_i,t_* is the time in years of the observation since baseline (with Years_*i*,0_ = 0 for every *i*), and AIBL*_i_* and HA*_i_* are indicators taking the value 1 for participants in the AIBL and HA groups, respectively, and 0 otherwise. Estimation was least squares for unbalanced panel data, so that no participants were excluded because of a missing observation. Heteroskedasticity-consistent standard errors clustered at the participant level were used to construct confidence intervals for estimates of β_1_ and β_2_.

The coefficients β_1_ and β_2_ give the mean changes per year in the outcome variable for the AIBL and HA participants, respectively. The difference β_2_ − β_1_ is the primary object of interest in this study, giving the difference between the HA and AIBL group mean changes per year in the outcome variable. The participant-specific time-invariant fixed effects α*_i_* control for all individual baseline characteristics, observable or otherwise, including age, sex, hearing loss, education, and pre-existing health conditions, among others.

#### Education

While the use of fixed effects can control for differences in cognition at baseline due to demographic characteristics of the participants, it does not address differences between the groups in cognitive trajectories over time. HA participants were, on average, more educated than AIBL participants (HA: 83.9%, AIBL: 72.5% with more than 12 years), so an additional three-way interaction analysis was used to investigate the effect of differences in education between the groups on trajectory of cognitive change. The specification was as follows:


(2) $$\begin{array}{l} Y_{i,t}=\alpha_{i}+\beta_{1}\;\left({\mathrm{AIBL}}_{i}\times {\mathrm{EducL}}_{i}\times {\mathrm{Years}}_{i,t}\right)\\ +\beta_{2}\;\left({HA}_{i}\times {\mathrm{EducL}}_{i}\times {\mathrm{Years}}_{i,t}\right)\\ +\beta_{3}\;\left({\mathrm{AIBL}}_{i}\times {\mathrm{EducH}}_{i}\times {\mathrm{Years}}_{i,t}\right)\\ +\beta_{4}\;\left({HA}_{i}\times {\mathrm{EducH}}_{i}\times {\mathrm{Years}}_{i,t}\right)+U_{i,t}\text{.} \end{array}$$

where EducL*i* equals 1 if the participant *i* has completed at most 12 years of education (0 otherwise), EducH*_i_* equals 1 if the participant has completed more than 12 years of education (0 otherwise), and the other variables are the same as in Equation (1). The coefficients β_1_ and β_3_ (β_2_ and β_4_) give the mean changes per year in the outcome variable for the AIBL (HA) participants with lower and higher levels of education, respectively. The differences β_2_ − β_1_ and β_4_ – β_3_ give the differences between the HA and AIBL group mean changes per year for the lower and higher levels of education, respectively.

#### Physical activity

As shown in Table 1, HA participants in this study had significantly higher mean levels of baseline physical activity (*p* < 0.05) than did AIBL participants, and Table 2 shows trends (albeit insignificant) for activity to increase over time for the HA participants and to decrease for the AIBL participants. Given evidence that cognition and physical activity may be expected to be positively correlated (Lautenschlager et al., 2008; Erickson et al., 2012; Bherer et al., 2013), a sensitivity analysis was carried out to determine whether this influenced the main results.

**Table 2:** Cognitive performance (raw scores) on the Cogstate Brief Battery (CSBB) at baseline, 18-, and 36-month follow-up for hearing aid and AIBL participants, and the Mini-Mental State Examination (MMSE) screening tool at baseline.

	HA participants			AIBL participants			HA versus AIBL (value of *p*)		
	Baseline	18 months	36 months	Baseline	18 months	36 months	Baseline	18 months	36 months
*Psychomotor function*									
*n*	160	61	54	102	49	18			
Mean	2.62	2.6	2.63	2.54	2.59	2.66	**0.000**	0.457	0.351
Median	2.6	2.6	2.6	2.5	2.6	2.6			
SD	0.1	0.1	0.1	0.1	0.1	0.1			
Min	2.4	2.4	2.4	2.4	2.4	2.5			
Max	2.9	2.8	3	2.8	2.8	3			
*Working memory*									
*n*	160	61	54	102	49	18			
Mean	2.96	2.93	2.95	2.71	2.87	2.95	**0.000**	0.173	0.956
Median	3	2.9	2.9	2.9	2.9	3			
SD	0.1	0.1	0.1	0.6	0.3	0.1			
Min	2.7	2.8	2.7	1.1	1.3	2.8			
Max	3.2	3.2	3.2	3.1	3.1	3.1			
*Attention*									
*n*	160	61	54	102	49	18			
Mean	2.79	2.78	2.78	2.74	2.77	2.8	**0.000**	0.883	0.366
Median	2.80	2.8	2.8	2.7	2.8	2.8			
SD	0.1	0.1	0.1	0.1	0.1	0.1			
Min	2.7	2.6	2.6	2.6	2.7	2.7			
Max	3	2.9	2.9	2.9	2.9	3.1			
*Visual learning*									
*n*	160	61	54	102	49	18			
Mean	0.97	0.97	0.98	1.01	1.01	1	**0.002**	**0.020**	0.469
Median	1	1	1	1	1	1			
SD	0.1	0.1	0.1	0.1	0.1	0.1			
Min	0.4	0.6	0.7	0.7	0.7	0.9			
Max	1.2	1.2	1.2	1.4	1.3	1.2			
*MMSE*									
*n*	160	0	0	99	0	0			
Mean	28.73			28.65			0.624		
Median	29			29					
SD	1.2			1.4					
Min	24			24					
Max	30			30					

Bold denotes significant differences (*p* < 0.05; significant at the 5% confidence level). The MMSE was administered only at baseline as a screening assessment for dementia. The Cogstate Brief Battery was used thereafter due to its lack of practice effects, high sensitivity to changes in cognitive performance, specificity for various cognitive functions, and non-auditory method of administration.

Since higher baseline levels of physical activity are already controlled for in Equations (1) and (2) by the individual fixed effects, the sensitivity analysis analyzed possible correlation between the *trajectory* of activity during the study with the *trajectory* of cognition. The regression function for cognition conditional on activity and HA/AIBL participation interacted with regression was derived in Equation (5) below allowing for the possibly bidirectional causality between cognition and activity, represented by the equations as follows:


(3) $$Y_{1,i,t}=\gamma_{1}\;Y_{2,i,t}+{\alpha_{1,i}}^{*}+{\beta_{1}}^{*}‘X_{i,t}+V_{1,i,t}$$

(4) $$Y_{2,i,t}=\gamma_{2}\;Y_{1,i,t}+{\alpha_{2,i}}^{*}+{\beta_{2}}^{*}‘X_{i,t}+V_{2,i,t}$$

where *Y*_1,*i*,*t*_ is physical activity, and *Y*_2,*i*,*t*_ is a cognitive outcome and

$\begin{array}{l} X_{i,t}=({\mathrm{AIBL}}_{i}\times {\mathrm{EducL}}_{i}\times {\mathrm{Years}}_{i,t}{,HA}_{i}\times {\mathrm{EducL}}_{i}\times \;{\mathrm{Years}}_{i,t},\\ \;{\mathrm{AIBL}}_{i}\times {\mathrm{EducH}}_{i}\times {\mathrm{Years}}_{i,t}{,HA}_{i}\times \;{\mathrm{EducH}}_{i}\times {\mathrm{Years}}_{i,t}) \end{array}$

contains the explanatory variables in Equation (2). The variables *V*_1,*i,t*_ and *V*_2,*i,t*_ comprise the other causal factors of activity and cognition, respectively, with var.(*V*_1,*i,t*_) = σ_1_^2^, var.(*V*_2,*i,t*_) = σ_2_^2^, and cov(*V*_1,*i,t*_, *V*_2,*i,t*_) = σ_12_. The potential positive relationship between activity and cognition suggests that γ_1_ ≥ 0, γ_2_ ≥ 0, and σ_12_ ≥ 0.

The potential bidirectional causality between *Y*_1,*i*,*t*_ and *Y*_2,*i*,*t*_ and potential correlation between *V*_1,*i,t*_ and *V*_2,*i,t*_ imply that these equations do not directly represent regression relationships. In particular, Equation (4) does not represent the regression *E*(*Y*_2,*i*,*t*_ | *Y*_1,*i*,*t*_, *X*_*i*,*t*_) that would be obtained from regressing cognition on physical activity and HA/AIBL participation interacted with education. Instead, routine derivations lead to the regression function


(5) $$E\;\left(Y_{2,i,t}|Y_{1,i,t},X_{i,t}\right)=\lambda Y_{1,i,t}+\left(\alpha_{2,i}-\lambda \alpha_{2,i}\right)+{\left(\beta_{2}-\lambda \beta_{1}\right)}^{'}X_{i,t}$$

where

λ = (γ_1_ σ_1_^2^ + (1 + γ_1_ γ_2_) σ_12_ + γ_2_ σ_2_^2^)/(σ_1_^2^ + 2 γ_2_ σ_12_ + γ_2_^2^ σ_2_^2^),

β_1_ = (β_1_^*^ + γ_2_ β_2_^*^)/(1 − γ_1_ γ_2_), β_2_ = (β_2_^*^ + γ_1_ β_2_^*^)/(1 − γ_1_ γ_2_),

α_1,*i*_ = (α_1,i_^*^ + γ_2_ α_2,i_^*^)/(1 − γ_1_ γ_2_), α_2,*i*_ = (α_2,i_^*^ + γ_1_ α_2,i_^*^)/(1 − γ_1_ γ_2_).

Equation (5) shows that the coefficients that would be obtained from the regression are, in general, a complicated and uninterpretable mixture of the coefficients of the cognition and activity Equations (3) and (4). For example, λ, the coefficient on activity, is a mixture of the parameters γ_1_, γ_2_, and σ_12_ which determine the pattern of bidirectional causality between cognition and activity. If λ = 0, then the sign restrictions γ_1_ ≥ 0, γ_2_ ≥ 0, and σ_12_ ≥ 0 imply that γ_1_ = γ_2_ = 0, so that Equations (3) and (4) are uncorrelated, the trajectory of cognition is not affected by the trajectory of activity, and hence β_1_ = β_1_^*^ and β_2_ = β_2_^*^. Practically this means that an insignificant estimate for λ in (5) implies that the coefficients on *X*_*i*,*t*_ in that equation may be interpreted as estimates of β_2_^*^ in (4). See Appendix for the derivation of Equation (5).

## Results

### Participants

Participants were 160 adults (49% female participants; mean [range] age 74 [67–87] years) with mild-to-severe HL (mean PTA4 of 31 dB HL) who chose to use HAs fitted after baseline assessment, and a comparative group of 102 participants of the AIBL study (55% female participants; mean [range] age 74 [67–85] years old) with untreated HL or normal hearing (mean PTA4 of 21dBHL). Table 1 shows demographic and audiometric characteristics of both groups, and numbers of participants assessed at each time interval. Only 2 HA users did not use English as their preferred spoken language at home. All AIBL participants used English as their preferred language. Data for a subset of the first participants to reach either or both follow-up points by end 2022 are compared, excluding data for 2 AIBL participants who did not attempt all subtests, one each at 18- and 36-month follow-up (Table 1). As this study is ongoing with continuous recruitment, most participants who were not assessed at these follow-up points had not dropped out of the study but had not yet reached these follow-up points and will be assessed in future. For the HA group, follow-ups totaled 61 and 54 participants at 18 and 36 months, respectively. For the AIBL group, follow-ups totaled 49 and 18 at these time points. There were no significant differences between the groups for age, sex, diabetes prevalence, falls, smoking, and depression (*p* > 0.05). At baseline and at follow-up, HA participants had significantly (*p* < 0.05) greater HL than AIBL participants (46% of AIBL participants had normal hearing at baseline) and were significantly (*p* < 0.05) more likely to have tertiary education, anxiety, and to be more physically active. See statistical analysis section for how these differences were controlled. Hearing data is missing for 11 AIBL participants at 18 months due to the inability to do in-person audiometry during COVID outbreaks. Education information was not given by 23 HA participants.

### Cognitive performance at baseline

Table 2 shows mean baseline and follow-up scores on the Cogstate Brief Battery subtests for both participant groups. At baseline, mean cognitive scores for the AIBL participants were significantly better than for the HA participants on all four subtests, despite significantly lower education.

### Hearing aid compliance and benefit at 36-month follow-up (HA participants)

At 36-month follow-up, HA participants demonstrated good device compliance and benefit despite relative isolation for approximately 2 years in COVID lockdowns. Table 3 shows objective HA usage (data logging) and benefit (speech perception). At 18-month follow-up, mean HA usage was 9.1 h/day, with usage dropping at 36 months after 18 months of successive COVID lockdowns to 7.9 h/day. Mean objective CVC word speech perception scores in quiet were very high at baseline (84.9% word; 94% phoneme) and improved at both follow-up points, although this was not statistically significant (likely due to a ceiling effect). SRT scores in noise improved significantly from baseline to 18 months (*p* = 0.017), with a non-significant decrease at 36 months.

**Table 3:** Treatment compliance and benefit: objective (data logging) data on hearing aid use and speech perception outcomes at baseline, 18 months, and 36 months.

	Baseline	18 months	36 months	*p*-values	
				Baseline versus 18 months	18 months versus 36 months
*HA usage (hours/day)*					
*n*		46	37		
Mean		9.07	7.93		0.110
Median		10	9		
S.D.		4.5	4.7		
Min		0	0.5		
Max		16	16		
*HA usage (%/day)*					
>90%		26.1	24.3		
60–90%		37	27		
30–60%		17.4	16.2		
<30%		19.6	32.4		
*CVC words (%)*					
*n*	157	56	44		
Mean	84.92	90.86	88.14	0.762	0.203
Median	92	94	90		
S.D.	16.2	9	10.4		
Min	8	64	52		
Max	100	100	100		
*CVC phonemes (%)*					
*n*	157	56	44		
Mean	94.03	96.67	95.39	0.274	0.092
Median	97	98	96.7		
S.D.	9.8	3.4	4.8		
Min	24.7	86	78		
Max	150	100	100		
*SRT*					
*n*	157	56	44		
Mean	0.22	−0.33	0.05	**0.017**	0.117
Median	−0.4	−0.4	−0.4		
S.D.	2.5	1.1	1.6		
Min	−2.7	−2.9	−2.7		
Max	18.7	2	4.6		

Bold denotes significant differences (*p* < 0.05; significant at the 5% confidence level). Increased CVC word and phoneme scores indicate improved performance in quiet listening conditions. As the SRT score measures the signal-to-noise ratio at which 50% of the key words are correct in noisy listening conditions, a decrease in SRT score represents improved performance.

### Cognitive performance at follow-up

**Figure 1:**
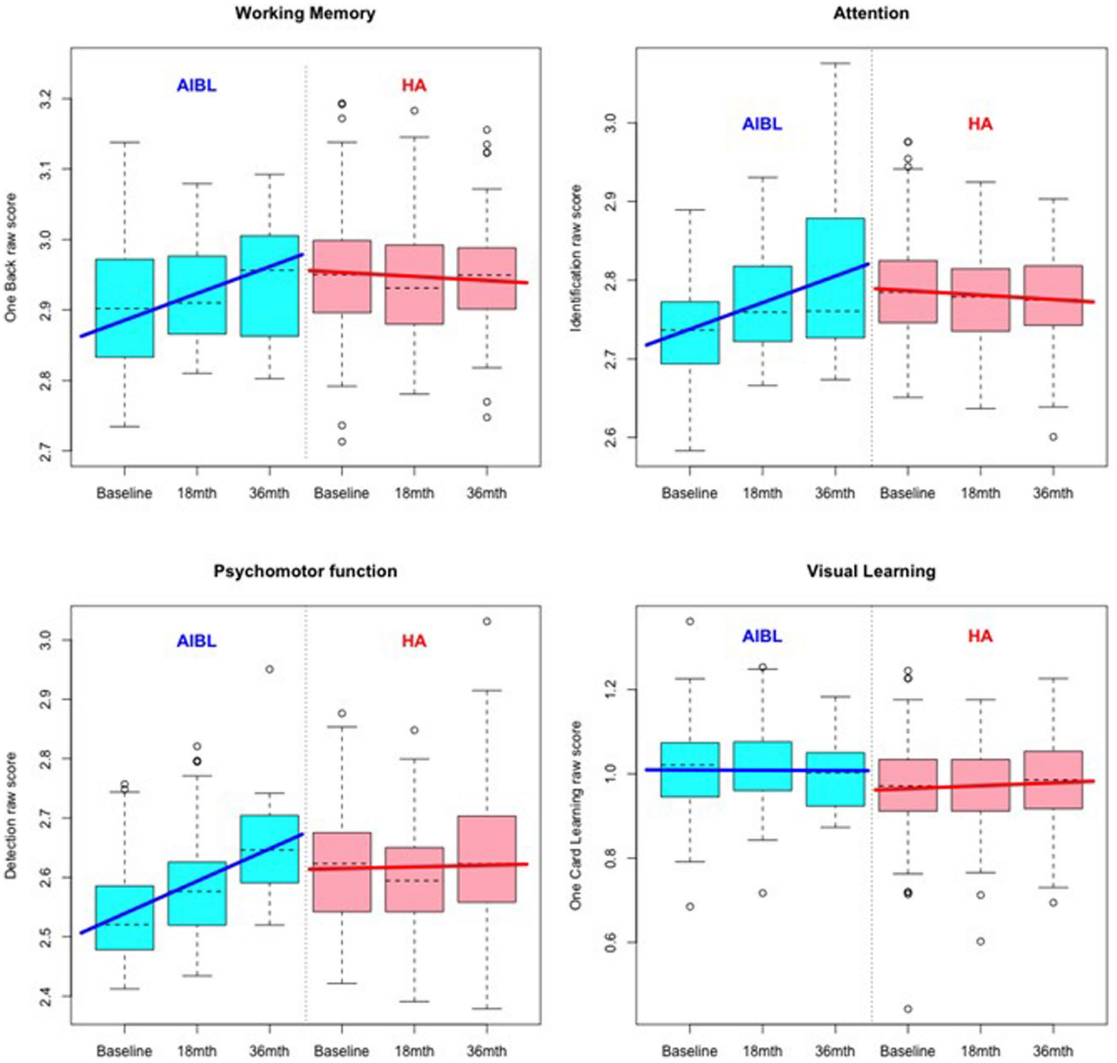
Comparative cognitive performance over 36 months for the HA and AIBL groups on the Cogstate Brief Battery. Scoring is based on speed and accuracy; therefore, increased scores indicate poorer performance. Relative to HA participants, scores for AIBL participants increased over time, while those for HA participants remained relatively stable.

A primary multiple regression analysis of comparative scores on the Cogstate Brief Battery used fixed effects to control for time invariant observed and unobserved participant characteristics at baseline. At follow-up, there were no longer any significant differences between group mean scores on cognitive subtests except on visual learning at 18 months. Comparative results for HA and AIBL participants at 18- and 36-month follow-up points are shown in Table 4 and in Figure 1. The primary outcome in Table 4 is given by β_1_ − β_2_, the difference between the HA and AIBL group mean changes per year across each dependent variable (subtest). The AIBL group showed significantly greater worsening of mean scores per year relative to the HA participants on all subtests except visual learning, where the trend was the same but not statistically significant. This occurred in conjunction with a significantly faster rate of HL over the follow-up period for the HA group (1.2 dB/year vs. 0.5 dB/year), although hearing in the AIBL group also declined significantly in the 18- to 36-month period. The AIBL group mean score declined by 3.1% of the baseline mean score per year on working memory, while the HA participants improved by 0.1% per year. On visual learning, the HA participants improved by 0.3% while the AIBL group declined by 0.8%. On attention, the HA group improved by 0.1% while the AIBL group declined by 0.8%, and on psychomotor function, the HA group declined by 0.4% while the AIBL group declined by 1.2%.

**Table 4:** Multiple regression analysis results of comparative cognitive scores on the Cogstate Brief Battery for the HA and AIBL groups at both 18- and 36-month follow-up.

Dependent variable	Working memory	Visual learning	Visual attention	Psychomotor function
*AIBL participants*				
β_1_	3.1	−0.8	0.8	1.2
95% C.I.	**(0.8, 5.3)**	(−2, 0.4)	**(0.4, 1.1)**	**(0.8, 1.6)**
*HA participants*				
β_2_	−0.1	0.3	−0.1	0.4
95% C.I.	(−0.3, 0.1)	(−0.5, 1.1)	(−0.2, 0.1)	**(0.1, 0.7)**
*HA-AIBL*				
β_2_ – β_1_	−3.2	1.1	−0.9	−0.8
95% C.I.	**(−5.4, −0.9)**	(−0.3, 2.5)	**(−1.2, −0.5)**	**(−1.3, −0.3)**
*R*^2^	0.13	0.015	0.177	0.171
Mean, dependent variable	2.9	1.0	2.8	2.6
S.D., dependent variable	0.4	0.1	0.1	0.1

See Equation (1). β_1_: average change per year for the AIBL participants, expressed as a percentage of the mean baseline score. β_2_: average change per year for the HA participants, expressed as a percentage of the mean baseline score. Subtests are scored for speed and errors, so lower scores indicate better performance. The visual learning subtest is reverse-scored, with increasing numbers indicating better performance. Bold denotes significant differences (*p* < 0.05; significant at the 5% confidence level). SD, standard deviation; CI, 95% confidence interval.

In terms of dementia outcomes, one HA participant was diagnosed with Lewy body dementia, and five AIBL participants were diagnosed with mild cognitive impairment by 36-month follow-up.

**Figure 2:**
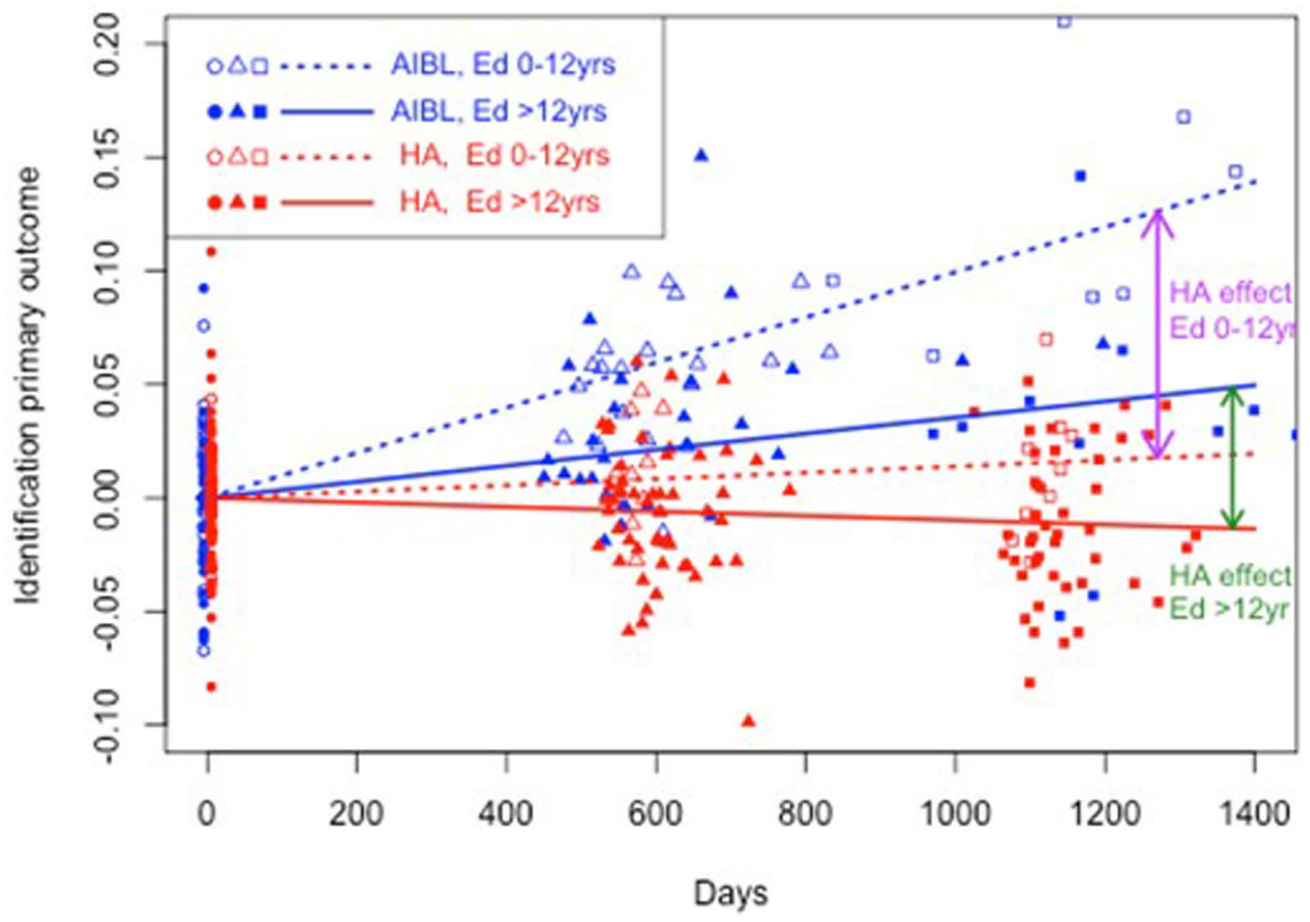
Estimated mean trajectories of cognitive change over 36 months, controlling for education, on the Cogstate Brief Battery visual attention subtest. Trajectories are net of individual specific characteristics that influence baseline cognitive performance. The x-axis shows days since baseline. The y-axis shows primary outcome raw scores. All baselines have a zero mean by construction as mean individual fixed effects have been subtracted out. AIBL participants are deteriorating significantly faster than HA participants (note reverse scoring means increasing scores indicate worse performance). After controlling for education, lower and higher educated participants in the HA group performed significantly better on visual attention than both lower and higher educated AIBL group participants.

**Figure 3:**
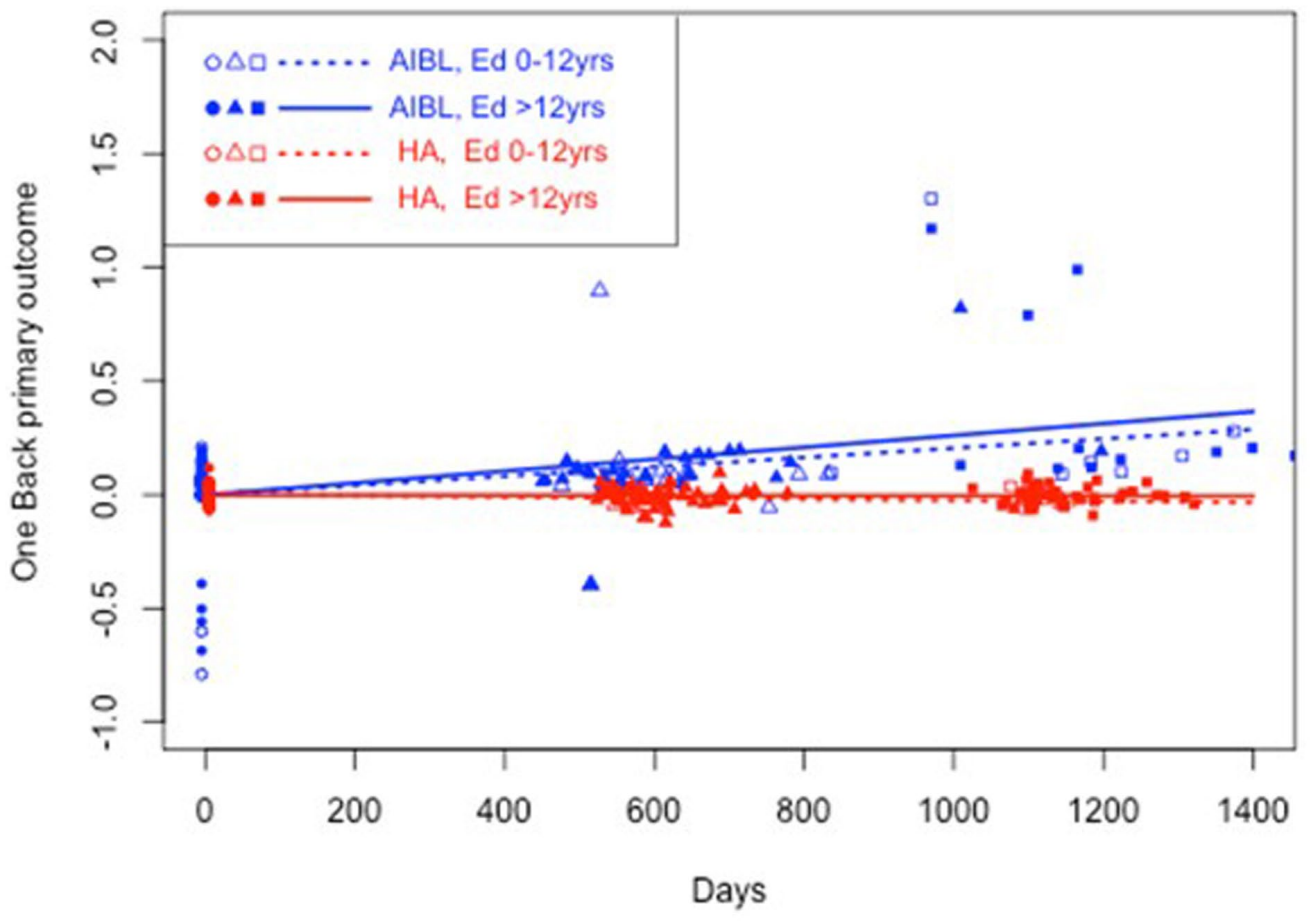
Estimated mean trajectories of cognitive change over 36 months, controlling for education, on the Cogstate Brief Battery working memory subtest. Trajectories are net of individual specific characteristics that influence baseline cognitive performance. The x-axis shows days since baseline. The y-axis shows primary outcome raw scores. All baselines have a zero mean by construction as mean individual fixed effects have been subtracted out. AIBL participants are deteriorating significantly faster than HA participants (note reverse scoring means increasing scores indicate worse performance). When education was controlled, higher educated HA participants performed significantly better than higher educated AIBL participants.

**Figure 4:**
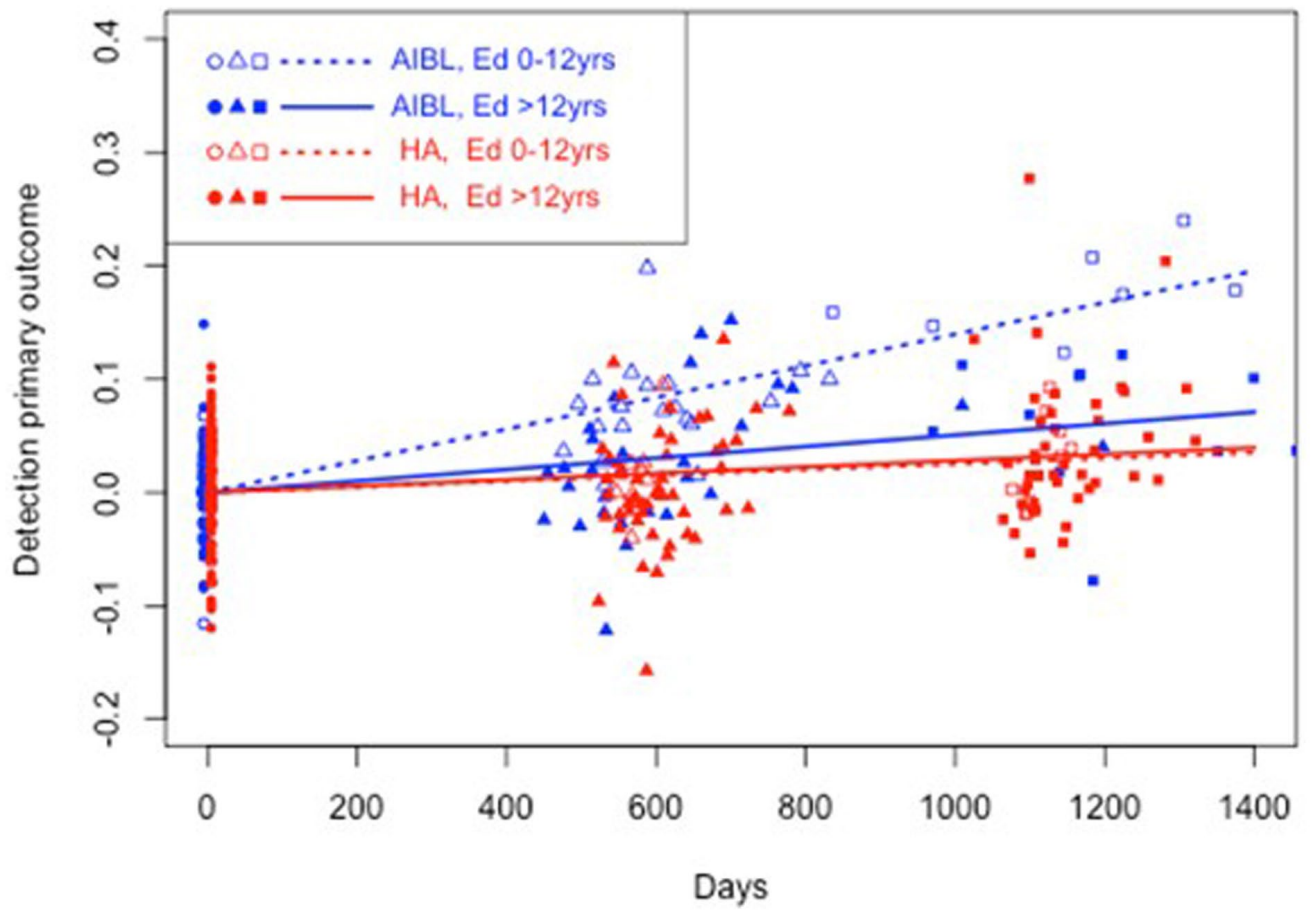
Estimated mean trajectories of cognitive change over 36 months, controlling for education, on the Cogstate Brief Battery psychomotor function subtest. Trajectories are net of individual specific characteristics that influence baseline cognitive performance. The x-axis shows days since baseline. The y-axis shows primary outcome raw scores. All baselines have a zero mean by construction as mean individual fixed effects have been subtracted out. AIBL participants are deteriorating significantly faster than HA participants (note reverse scoring means increasing scores indicate worse performance). After controlling for education, lower educated participants in the HA group performed significantly better on psychomotor function than lower educated AIBL group participants.

Table 5 shows the results of a three-way interaction sensitivity analysis to control the effect of differences in education on the trajectory of cognitive change for the HA and AIBL groups. When interactions with education were included, HA participants still performed significantly better than AIBL participants on visual attention regardless of educational status. On working memory, higher educated HA participants performed significantly better than higher educated AIBL participants (0 vs. 3.3% decline per year). On psychomotor function, lower educated HA participants performed significantly better (an insignificant 0.4% per year decline) than did lower educated AIBL participants (a significant 2.0% per year decline). Higher educated HA participants declined significantly but at a (not significantly) lower rate (0.4%) than for higher educated AIBL participants (0.7%). There were no significant changes in cognitive outcomes for visual learning in this analysis. Figures 2–4 illustrate outcomes for the three subtests on which there was an effect of education on cognitive trajectory.

**Table 5:** Three-way interaction sensitivity analysis to control for the effect of education on trajectory of cognitive change: HA versus AIBL groups.

	Working memory	Visual learning	Visual attention	Psychomotor function
*AIBL, lower education*				
β_1_	2.6	−0.1	1.3	2.0
95% C.I.	(−0.8, 6)	(−1.8, 1.5)	**(0.8, 1.8)**	**(1.5, 2.5)**
*HA, lower education*				
β_2_	−0.3	1.1	0.2	0.4
95% C.I.	(−0.7, 0.1)	(−1.5, 3.7)	(−0.2, 0.5)	(−0.3, 1.0)
*AIBL, higher education*				
β_3_	3.3	−1.2	0.5	0.7
95% C.I.	**(0.4, 6.3)**	(−2.8, 0.4)	**(0.1, 0.8)**	**(0.2, 1.2)**
*HA, higher education*				
β_4_	0.0	0.1	−0.1	0.4
95% C.I.	(−0.2, 0.1)	(−0.7, 1)	(−0.3, 0.1)	**(0, 0.8)**
*HA, lower education – AIBL, lower education*				
β_2_ – β_1_	−2.9	1.2	−1.1	−1.6
95% C.I.	(−6.4, 0.5)	(−1.8, 4.3)	**(−1.8, −0.5)**	**(−2.4, −0.8)**
*HA, higher education – AIBL, higher education*				
β_4_ – β_3_	−3.4	1.4	−0.6	−0.3
95% C.I.	**(−6.4, −0.4)**	(−0.4, 3.2)	**(−1, −0.2)**	(−0.9, 0.3)
*R*^2^	0.132	0.023	0.231	0.208
Mean, dependent variable	2.9	1	2.8	2.6
S.D., dependent variable	0.4	0.1	0.1	0.1

See Equation (2). β_1_: average change per year for lesser educated AIBL participants, expressed as a percentage of the mean baseline score. β_2_: average change per year for lesser educated HA participants, expressed as a percentage of the mean baseline score. β_3_: average change per year for higher educated AIBL participants, expressed as a percentage of the mean baseline score. β_4_: average change per year for higher educated HA participants, expressed as a percentage of the mean baseline score. Bold denotes significant differences (*p* < 0.05; significant at the 5% confidence level). SD, standard deviation; CI, 95% confidence interval.

Table 6 shows the results of a sensitivity analysis to control for the effect of differences in physical activity between the HA and AIBL groups on trajectory of cognitive change. The estimate of λ in Equation (5) was insignificant for every outcome, indicating that there were no effects of physical activity on cognitive performance.

**Table 6:** Sensitivity analysis to control for the effect of physical activity on cognitive change: HA versus AIBL groups.

	Working memory	Visual learning	Attention	Psychomotor function
*AIBL, lower education*				
β_1_	2.8	−0.1	1.3	1.9
95% C.I.	(−0.7, 6.2)	(−1.7, 1.6)	**(0.7, 1.8)**	**(1.4, 2.5)**
	2.8	−0.1	1.3	1.9
*HA, lower education*				
β_2_	−0.3	1.1	0.2	0.4
95% C.I.	(−0.7, 0.1)	(−1.5, 3.7)	(−0.2, 0.6)	(−0.3, 1)
*AIBL, higher education*				
β_3_	3.5	−1.1	0.4	0.7
95% C.I.	**(0.5, 6.5)**	(−2.7, 0.4)	**(0.1, 0.8)**	**(0.2, 1.2)**
*HA, higher education*				
β_4_	0	0.2	−0.1	0.4
95% C.I.	(−0.2, 0.2)	(−0.7, 1)	(−0.3, 0)	**(0, 0.8)**
*HA, lower education – AIBL, lower education*				
β_2_ – β_1*_	−3.1	1.2	−1.1	−1.6
95% C.I.	(−6.6, 0.4)	(−1.9, 4.2)	**(−1.8, −0.4)**	**(−2.4, −0.7)**
*HA, higher education – AIBL, higher education*				
β_4_ – β_3_	−3.5	1.3	−0.6	−0.3
95% C.I.	**(−6.6, −0.5)**	(−0.5, 3.1)	**(−1, −0.2)**	(−0.9, 0.3)
*Physical activity*				
λ	0.42	0.19	−0.09	−0.1
95% C.I.	(−0.41, 1.25)	(−0.25, 0.63)	(−0.21, 0.03)	(−0.27, 0.06)
*R*^2^	0.147	0.027	0.248	0.22
Mean, dependent variable	2.9	1	2.8	2.6
S.D., dependent variable	0.4	0.1	0.1	0.1

See Equation (5). Y*_i,t_* = α*_i_* + β_1_ (AIBL*_i_* × EducL*_i_* × Years*_i,t_*) + β_2_ (HA*_i_* × EducL*_i_* × Years*_i,t_*) + β_3_ (AIBL*_i_* × EducH*_i_* × Years*_i,t_*) + β_4_ (HA*_i_* × EducH*_i_* × Years*_i,t_*) + λ Activity*_i,t_* + U*_i,t_*. Bold denotes significant differences (*p* < 0.05; significant at the 5% confidence level).

Although the purpose of this study was to compare the cognitive performance of HA users with a representative sample of untreated older community-living adults, there was disparity between the groups in terms of HL, with almost half of the AIBL group with no HL. Therefore, a further sensitivity analysis was conducted including only AIBL participants with HL. Table 7 shows only small changes in estimates and the same outcome as for previous analyses.

**Table 7:** Sensitivity analysis of cognitive performance outcomes for HA vs AIBL participants with hearing loss only.

**Dependent variable**	**Working memory**	**Visual learning**	**Visual** **Attention**	**Psychomotor function**	**Better Ear PTA**^**a**^	**Log Activity**
*AIBL participants*						
β_1_	4	−0.6	0.5	1.1	2.5	−3.2
95% C.I.	**(0.1, 7.9)**	(−2, 0.8)	(−0.1, 1.1)	**(0.7, 1.5)**	**(0.6, 4.3)**	(−14.4, 7.9)
*HA participants*						
β_2_	−0.1	0.3	−0.1	0.4	4	0.3
95% C.I.	(−0.3, 0.1)	(−0.5, 1.1)	(−0.2, 0.1)	**(0.1, 0.7)**	**(2.9, 5.1)**	(−0.5, 1.2)
*HA-AIBL*						
β_2_ – β_1_	−4.1	0.9	−0.6	−0.7	1.5	3.6
95% C.I.	**(−8, −0.2)**	(−0.7, 2.6)	**(−1.2, 0)**	**(−1.2, −0.2)**	(−0.6, 3.7)	(−7.6, 14.7)
*R*^2^	0.165	0.008	0.056	0.124	0.282	0.01
Mean, dependent variable	2.9	1.0	2.8	2.6	30.4	7.5
S.D., dependent variable	0.3	0.1	0.1	0.1	8.5	2.2

Equation: Y_i,t_ = α_i_ + β_1_ AIBL_i_*Years_i,t_ + β_2_ HA_i_*Years_i,t_ + U_i,t_ where t = 0,1,2: baseline, 18 months, 36 months respectively. Y_i,t_: outcome for participant i at time t. α_i_: participant fixed effect. AIBL_i_=1 if participant i is in AIBL group. HA_i_=1 if participant i is in HA group. Years_i,t_: time in years since baseline (Years_i,0_ = 0). In the table: β_1_: average change per year for AIBL participants, expressed as a percentage of the mean baseline score. β_2_: average change per year for HA participants, expressed as a percentage of the mean baseline score. β_2_ – β_1_: difference between HA and AIBL average changes per year, expressed as a percentage of the mean baseline score. Bold denotes significant differences (*p* < 0.05; significant at the 5% confidence level).

## Discussion

This prospective longitudinal cohort comparative study addresses several significant methodological limitations in the current evidence regarding the effects of HAs on cognitive decline, with the results suggesting that hearing intervention with HAs may delay cognitive decline. At baseline, cognitive scores were significantly poorer for the HA group across the assessment battery but not at follow-up. Comparatively, after 3 years of device use, the HA group showed overall stability in cognitive performance while the AIBL group declined significantly, despite having significantly less HL. The intervention and control groups differed significantly at baseline on education and level of physical activity, and these differences were controlled in statistical analyses. When education effects on cognitive trajectory were controlled, the HA group still performed significantly better overall in comparison to the AIBL group. Differing levels of physical activity had no effect on comparative cognitive outcomes. Hearing for the AIBL group also declined significantly in the 18- to 36-month follow-up period, but the AIBL group participants did not use HAs to address this. Although the purpose of this study was to compare cognitive performance of HA users with an untreated group representative of the older adult community, a final sensitivity analysis, including only AIBL participants with HL, addressed the difference in HL between the group. Even when AIBL participants without HL were excluded, the outcome remained the same. If there were to be any difference in outcomes between the whole AIBL group and a subset of this group with untreated HL only, we might expect the AIBL/HL group to be performing more poorly than the AIBL group as a whole. The finding that the HA group with HL is doing better than the AIBL group as a whole may in fact be a stronger result than finding the HA group is doing better than the AIBL/HL group. This beneficial outcome of HA use is interesting given the HA group had more risk factors for cognitive decline than did the AIBL group (i.e., poorer cognitive scores at baseline, greater HL, anxiety, and depression), although greater education may have ameliorated some of this risk. These results suggest that HA use in older adults with HL may be a useful intervention for slowing cognitive decline.

Estimates of cognitive decline of fluid skills on standardized tests of cognitive ability (e.g., processing speed, working memory, and long-term memory) derived from normative data are close to 1% per year in an approximately linear pattern with age, with little difference reported between male and female participants(Salthouse, 2010). The same patterns of cognitive aging are reported in well-replicated large-scale studies that represent the prototypical cognitive aging profile of the general population (Schaie, 2005; Salthouse, 2010; Salthouse, 2019). However, older adults who have untreated HL are reported to decline cognitively at 2–5 times the rate of adults without HL (Lin et al., 2013; Livingston et al., 2020). In this study, the AIBL group declined at the higher rate predicted for people with untreated HL on working memory (3%), and at approximately the same rate as the general population aged over 50 years (1% per year) on the other measures, while the HA group showed no significant decline except on psychomotor function, where the decline per year was less than that expected in the general population.

It is interesting to compare these results with those of the recent ACHIEVE study, which had the same length of follow-up, and reported an effect of HA use in a secondary analysis only for ARIC study HA user participants who were at higher risk of cognitive decline (Lin et al., 2023). Although the ARIC subgroup showed a 48% reduction in rate of decline, they did not demonstrate cognitive stability as did the HA users in this study. While the ACHIEVE investigators suggested that the slow rate of cognitive decline observed in their *de novo* cohort may have limited the effect of hearing intervention to reduce this rate of decline in only a 3-year follow-up period, this was not the case in the current study. The HA cohort in the ENHANCE study was likely at lower risk of cognitive decline than the ACHIEVE ARIC group as the ARIC group had more risk factors (i.e., age, female sex, lower education, greater prevalence of diabetes, hypertension, and living alone). However, a significant benefit to cognition was still seen in the ENHANCE study from hearing intervention in terms of both cognitive stability for three cognitive domains and a lower rate of decline than would be expected in the general population on psychomotor function. Possible explanations for the variance in outcomes could include differences in the sensitivity of cognitive assessment measures used between the studies (the CSBB is highly sensitive to small changes in function and has low practice effects that resolve quickly; Collie et al., 2003; Falleti et al., 2006) and mode of test administration, with participants in the ENHANCE study assessed visually and not required to process test instructions through audition only (or to use audition plus written information), causing likely greater cognitive load than for visual administration only. ACHIEVE outcomes may also have been negatively impacted by poorer HA use than in the ENHANCE study. While data logging showed average HA use of 9.1 and 7.9 h per day at 18 and 36 months, respectively, for the current study, ACHIEVE participants self-reported 7–8 h use per day, slightly less. As self-report is well documented to exaggerate device use by between 1 and 2 h per day (Laplante-Lévesque et al., 2014; Solheim and Hickson, 2017), actual ACHIEVE participant HA usage may have been only 5–6 h/day, potentially 3–4 h per day less than for ENHANCE participants. Over a 3-year period, this would amount to significantly less exposure to the hearing intervention. Of note is that both studies began prior to and were conducted throughout the COVID pandemic, suffering negative effects of COVID-related lockdowns. These were extreme for the ENHANCE study in Australia, with almost 2 years of consecutive lockdowns and a likely related drop in HA use observed at 18-month follow-up, as participants were mostly unable to leave their homes to socialize. As the ACHIEVE study was conducted across multiple states of the US, each of which managed COVID differently, there were likely variable effects on data collection and outcomes dependent on site/state. There were also differences between the studies in how data collection was conducted during this period, with the ACHIEVE study reducing its cognitive battery to six tests and conducting assessments over the phone, and the ENHANCE study continuing to administer the complete cognitive battery via laptops delivered to the homes of participants.

It is well-reported that there is an approximately linear trajectory of decline with age in the absence of neuropathological disease (Schaie, 2005; Salthouse, 2010) which education does not affect. Although related to cognitive performance, education is not related to the rate of cognitive decline (Zahodne et al., 2011; Clouston et al., 2019), with pre-morbid intelligence established as the more powerful determinant of incident dementia (brain reserve theory; Satz, 1993; Schmand et al., 1997). However, in the presence of pathology, a risk factor for cognitive decline is lower education level (Clouston et al., 2019). Possible effects of baseline differences between the groups in this study were therefore controlled using fixed effects, along with the effects of education differences between the groups on cognitive trajectories controlled in sensitivity analyses. Although the AIBL group was less well educated, they had overall higher mean cognitive scores at baseline, which possibly illustrates the known association between cognitive decline and age-related HL for the HA group. Overall, the AIBL group showed significantly greater decline than did the HA group for one or both levels of education.

Significant differences in levels of self-reported physical activity (as measured on the IPAQ; Craig et al., 2003) between the HA and AIBL groups were noted at baseline and at each follow-up point, with HA users consistently more active than AIBL participants. There were also trends toward decline in activity over time for the AIBL participants and toward increased activity for the HA participants. Despite epidemiological evidence supporting a positive correlation between cognitive performance and higher levels of physical activity (Yaffe et al., 2001; Lautenschlager et al., 2008; Sattler et al., 2011; Buchman et al., 2012), a sensitivity analysis showed no evidence of an association between changes in physical activity during the 3-year follow-up period and cognitive trajectory in this study. Although reviews of the evidence have concluded exercise is associated with a reduced risk of dementia (Erickson et al., 2012; Bherer et al., 2013; Livingston et al., 2017), studies of physical activity are complicated, and outcomes suggest the potential for reverse causation and risk reduction, as noted by the Lancet Commission on dementia (2020). As found by Sattler et al. (2011), measurements of motor coordination and physical fitness (usually related to moderate-to-vigorous exercise) may be better predictors of cognitive decline than self-rated physical activity in everyday life. It has also been noted that self-reported data on physical activity often only correlate at low-moderate levels with objective measures of daily physical activity (Westerterp, 2009) and do not capture non-intentional low-intensity activities such as fidgeting and pacing. These measures also suffer from social desirability bias, with frequently artificially inflated reports (Erickson et al., 2012). The null finding for physical activity could also be related to the fact that this result is related to changes in activity in only a 3-year period since baseline. It remains unclear over what period exercise/physical activity must be sustained, and how close to the time of risk this must occur to reduce dementia risk (Livingston et al., 2020).

The HA group in this study had a significantly higher prevalence of anxiety than did the AIBL group at baseline. Both anxiety and depression are associated with an increased risk of dementia (Byers and Yaffe, 2011; Becker et al., 2018; Santabárbara et al., 2019; Kuring et al., 2020; Oh et al., 2020). However, anxiety and depression are both prodromal features, and symptoms, of dementia; as cognition declines, there will likely be greater prevalence of anxiety and depression. It is therefore not possible to disentangle the effects of cognition on mental health or mental health on cognition in this dataset. For this reason, no sensitivity analyses were conducted to investigate the effects of these outcomes on cognitive trajectories. Furthermore, despite significantly worse mental health, the HA participants performed better than the AIBL participants.

The body of evidence on the effects of HA use on cognitive decline is growing, with many cross-sectional and longitudinal studies suggesting that HA use is associated with better cognitive performance (Deal et al., 2015; Castiglione et al., 2016; Karawani et al., 2018; Sarant et al., 2020). However, conflicting outcomes are reported, both in terms of which cognitive functions improve (e.g., significant improvement has been reported for executive function in two studies; Castiglione et al., 2016; Sarant et al., 2020) but not in a third (Phillips et al., 2022) and in terms of overall outcomes, with several studies finding no association between HA use and improved cognitive performance (Valentijn et al., 2005; van Hooren et al., 2005; Lin et al., 2013). As mentioned earlier, recent systematic reviews (Sanders et al., 2021; Yang et al., 2022; Yeo et al., 2022) have disagreed on the effect of HA use on cognition, and the World Health Organization (WHO) currently rates the quality of the current evidence as ‘very low’, with current WHO guidelines for risk reduction of cognitive decline and dementia stating ‘there is insufficient evidence to recommend the use of HAs to reduce the risk of cognitive decline and/or dementia’ (WHO, 2019). In addition to the methodological issues discussed earlier re objective and appropriate measurement of relevant outcomes, a further limitation of the existing evidence is the use of cognitive function vs. dementia as the primary outcome in observational studies of healthy adults (Brewster et al., 2022) as this constrains our ability to determine the effects of hearing intervention on the risk for dementia and to generalize existing evidence to older adults with pre-existing cognitive impairment. Given the short follow-up periods of most studies to date (6–18 months), this stage of cognitive decline is rarely reached; however, with longer follow-up in studies of initially healthy older adults, such as the current study and the AIBL study, this should be achievable.

A major strength of this study is the visual presentation of cognitive assessments to avoid confounding of cognitive data by HL. In contrast to the pattern of missing data observed in the ARIC study, with greater missingness associated with degree of HL on all auditory-only tests and some non-auditory only tests (Deal et al., 2021), data for only two AIBL participants in this study are not included in this dataset due to missingness for one subtest (one had no HL and the other a mild HL at 36-month follow-up only). Further strengths are a comparative group, a longer follow-up period than for most studies, and objective measurement of HL, HA use, and speech perception benefit from hearing intervention. An added strength of this study is that participants completed their cognitive assessments alone, and de-identified results were uploaded automatically to the remote Cogstate platform for automated analysis, eliminating any tester bias on results. Although this is an observational study, we were able to control for several of the conceivable factors other than HA use that may have influenced cognition over the 3-year trajectory using fixed effects and further sensitivity analyses for important factors such as HL, education, and physical activity. Although observational studies can only demonstrate associations, the quality of evidence they yield grows with the quality of controls.

This study has limitations. Generalizability of these results is currently limited by small sample sizes, particularly at 36-month follow-up, the result of COVID-related lockdowns over most of 2020–2021. Larger samples would provide more precise estimates of the true effect size of HA use on cognitive decline over time. There is likely participant selection bias, as although both groups chose to participate in research studies, the HA group chose to use HAs and the AIBL participants with HL chose not to do so or were unaware of their HL. Given this, HA and AIBL participants could have differed regarding characteristics such as negative self-attribution and other self-management strategies of health conditions, although all participants were assessed regularly by audiologists, and all had access to audiological care. Although baseline characteristics and several major risk factors for cognitive decline such as age and medical conditions did not significantly differ between the groups or were controlled, the groups may have differed in other unmeasured characteristics. As this study was not a RCT, it was not possible to control differences in cognitive trajectory associated with unmeasured baseline characteristics. However, the fact that the AIBL participants were more likely to have better hearing, better baseline cognitive performance, less depression, and less anxiety makes it more likely this group is a plausible indicator of the cognitive trajectory the HA participants may have followed if they had not received hearing intervention, and suggests that the direction of bias for the AIBL group was likely toward that of less decline than the HA group, given its known characteristics and risk factors for cognitive decline. However, the direction of bias with respect to unmeasured participant characteristics is unknown. Further possible methodological limitations could include self-report of dementia outcome, heterogeneity in the HAs themselves, variance in fitting appropriateness, and the amount of device use between individuals, although these results should be interpreted as means over variations in these characteristics across these participants. Future analyses with larger sample sizes may estimate means conditional on such characteristics of HA implementation.

## Conclusion

Addressing hearing loss may be an important public health strategy for reducing or delaying the global burden of dementia. This prospective longitudinal cohort comparative study addresses several significant methodological limitations in previous studies regarding the effects of HAs on cognitive decline, with the results suggesting that hearing intervention may delay cognitive decline. Further recruitment and follow-up are ongoing to address sample size and investigate the size and duration of any longer-term effects on cognitive performance. The outcomes of this study and those of the recent ACHIEVE study add to a growing body of evidence that suggests hearing intervention, currently very underused and with no medical risk, may be an effective method of reducing or delaying (but likely not preventing) cognitive decline. Better hearing and communication abilities will also promote healthy aging and better quality of life. The potential clinical implications of this evidence should be considered. Further studies of populations at risk for dementia that address the methodological limitations outlined earlier are required to improve our understanding of the association between HL and dementia. Future research should assess not only cognitive performance but also incident dementia to facilitate estimation of dementia conversion rates that may be attributable to HL. They should also include assessment of functional outcomes of HA use and investigation of any relationship between these and cognitive performance and any influence on delay of dementia onset. Further challenges may include determining when in the lifespan to begin monitoring hearing, when to intervene, and establishing whether HA use is a cost-effective method of dementia delay that could be implemented at scale as a public health strategy. If this is the case, interdisciplinary collaboration, including medical referral for hearing care and availability of this, will be key contributing factors to improved health span and quality of life for older adults in future.

## Data availability statement

The datasets presented in this article are not available as the ethical consent provided by participants does not permit use of the data beyond the current study. The publication of this data does not compromise the anonymity of the participants or breach local data protection laws.

## Ethics statement

The studies involving humans were approved by the University of Melbourne Behavioral and Social Sciences Human Ethics Sub-Committee (Ethics ID: 1646925). The studies were conducted in accordance with the local legislation and institutional requirements. The participants provided their written informed consent to participate in this study in accordance with the Declaration of Helsinki.

## Author contributions

JS: Conceptualization, Formal analysis, Funding acquisition, Investigation, Methodology, Project administration, Supervision, Visualization, Writing – original draft, Writing – review & editing. PB: Data curation, Software, Writing – review & editing. AS: Resources, Writing – review & editing. CF: Data curation, Investigation, Writing – review & editing. DH: Conceptualization, Formal analysis, Methodology, Visualization, Writing – original draft, Writing – review & editing.
