# Supplementary material for: ENHANCE: a comparative prospective longitudinal study of cognitive outcomes after 3 years of hearing aid use in older adults
Source: Front Aging Neurosci. 2024 Jan 31;15:1302185. doi: 10.3389/fnagi.2023.1302185 (PMC10864469; doi:10.3389/fnagi.2023.1302185)
Supplement: Supplementary file 1 [file Data_Sheet_1.pdf]

Appendix : Derivation of Equation (5)

Equations (3) and (4) are written

$$\Gamma Y_{i,t} = \alpha_i^* + B^* X_{i,t} + V_{i,t}$$

where

$$\Gamma = \begin{pmatrix} 1 & -\gamma_1 \\ -\gamma_2 & 1 \end{pmatrix}, \quad B^* = \begin{pmatrix} \beta_1^{*\prime} \\ \beta_2^{*\prime} \end{pmatrix}, \quad \Sigma = \mathrm{var}(V_{i,t}) = \begin{pmatrix} \sigma_1^2 & \sigma_{1,2} \\ \sigma_{1,2} & \sigma_2^2 \end{pmatrix}.$$

Solve for $Y_{i,t}$:

$$\begin{aligned} Y_{i,t} &= \Gamma^{-1}\alpha_i^* + \Gamma^{-1}B^* X_{i,t} + \Gamma^{-1}V_{i,t} \\ &= \alpha_i + BX_{i,t} + U_{i,t}, \end{aligned}$$

where

$$\alpha_i = \begin{pmatrix} \alpha_{1,i} \\ \alpha_{2,i} \end{pmatrix}, \quad B = \begin{pmatrix} \beta_1' \\ \beta_2' \end{pmatrix}, \quad \Gamma^{-1} = \frac{1}{1-\gamma_1\gamma_2}\begin{pmatrix} 1 & \gamma_2 \\ \gamma_1 & 1 \end{pmatrix}.$$

The second equation represents the usual regression of cognition on $X_{i,t}$ in Table 5. Thus

$$B = \frac{1}{1-\gamma_1\gamma_2}\begin{pmatrix} \beta_1^{*\prime} + \gamma_2\beta_2^{*\prime} \\ \gamma_1\beta_1^{*\prime} + \beta_2^{*\prime} \end{pmatrix} = \begin{pmatrix} \beta_1' \\ \beta_2' \end{pmatrix}.$$

The variance matrix of $U_{i,t}$ can be written

$$\begin{aligned} \mathrm{var}(U_{i,t}) =& \Gamma^{-1}\Sigma\Gamma^{-1\prime} \\ =& \frac{1}{(1-\gamma_1\gamma_2)^2}\begin{pmatrix} \sigma_1^2 + 2\gamma_2\sigma_{1,2} + \gamma_2^2\sigma_2^2 & \gamma_1\sigma_1^2 + (1+\gamma_1\gamma_2)\sigma_{1,2} + \gamma_2\sigma_2^2 \\ \gamma_1\sigma_1^2 + (1+\gamma_1\gamma_2)\sigma_{1,2} + \gamma_2\sigma_2^2 & \gamma_1^2\sigma_1^2 + 2\gamma_1\sigma_{1,2} + \sigma_2^2 \end{pmatrix} \\ =& \Omega = \begin{pmatrix} \omega_1^2 & \omega_{1,2} \\ \omega_{1,2} & \omega_2^2 \end{pmatrix} \end{aligned}$$

The decomposition of Ω is

$$\begin{pmatrix} \omega_1^2 & \omega_{1,2} \\ \omega_{1,2} & \omega_2^2 \end{pmatrix} = \begin{pmatrix} 1 & 0 \\ \frac{\omega_{1,2}}{\omega_1^2} & 1 \end{pmatrix}\begin{pmatrix} \omega_1^2 & 0 \\ 0 & \omega_2^2 - \frac{\omega_{1,2}^2}{\omega_2^2} \end{pmatrix}\begin{pmatrix} 1 & 0 \\ \frac{\omega_{1,2}}{\omega_1^2} & 1 \end{pmatrix}' = LDL'$$

where

$$L^{-1} = \begin{pmatrix} 1 & 0 \\ \frac{\omega_{1,2}}{\omega_1^2} & 1 \end{pmatrix}^{-1} = \begin{pmatrix} 1 & 0 \\ -\frac{\omega_{1,2}}{\omega_1^2} & 1 \end{pmatrix}$$

Then

$$L^{-1}Y_{i,t} = L^{-1}\alpha_i + L^{-1}BX_{i,t} + L^{-1}U_{i,t}$$

or

$$\begin{aligned} Y_{1,i,t} &= \alpha_{1,i} + \beta_1' X_{i,t} + U_{1,i,t} \\ Y_{2,i,t} &= \frac{\omega_{1,2}}{\omega_1^2}Y_{1,i,t} + \left(\alpha_{2,i} - \frac{\omega_{1,2}}{\omega_1^2}\alpha_{1,i}\right) + \left(\beta_2 - \frac{\omega_{1,2}}{\omega_1^2}\beta_1\right)' X_{i,t} + \left(U_{2,i} - \frac{\omega_{1,2}}{\omega_1^2}U_{1,i}\right). \end{aligned}$$

Note the constructed orthogonality of these two equations, so that taking the conditional expectation of the latter equation given $Y_{1,i,t}$ and $X_{i,t}$ gives form of the regression equation for $Y_{2,i,t}$ on $Y_{1,i,t}$ and $X_{i,t}$ with individual fixed effects.
